# Analysis of Key Genes Responsible for Low Urea Production in *Saccharomyces cerevisiae* JH301

**DOI:** 10.3389/fmicb.2022.894661

**Published:** 2022-04-26

**Authors:** Zhangcheng Liang, Hao Su, Xiangyun Ren, Xiaozi Lin, Zhigang He, Xiangyou Li, Yan Zheng

**Affiliations:** ^1^Institute of Agricultural Engineering Technology, Fujian Academy of Agricultural Sciences, Fuzhou, China; ^2^Fujian Key Laboratory of Agricultural Products (Food) Processing, Fuzhou, China; ^3^Fujian Pinghuhong Biological Technology Co., Ltd., Fuzhou, China

**Keywords:** key gene, whole genome sequencing, comparative genomic analysis, Hongqu Huangjiu, *Saccharomyces cerevisiae*, low urea production

## Abstract

There is a potential safety risk with ethyl carbamate (EC) in Hongqu Huangjiu production; 90% of the EC in rice wine is produced by the reaction of the urea with the alcohol of *Saccharomyces cerevisiae*. In our previous experiments, we screened and obtained a *S. cerevisiae* strain JH301 that offered low urea production. However, the key genes responsible for low urea production of strain JH301 remain unclear. Here, the whole genome sequencing of *S. cerevisiae* strain JH301 was accomplished via a next-generation high-throughput sequencing and long-read sequencing technology. There are six main pathways related to the urea metabolism of strain JH301 based on KEGG pathway mapping. Three species-specific genes are related to the urea metabolism pathways and were found in comparative genome analysis between strains JH301 and S288c during Hongqu Huangjiu production for the first time. Finally, the *ARG80* gene was found to be likely a key gene responsible for low urea production of *S. cerevisiae* strain JH301, as determined by PCR and qRT-PCR check analyses from DNA and RNA levers. In conclusion, the results are useful for a scientific understanding of the mechanism of low urea production by *Saccharomyces cerevisiae* during Hongqu Huangjiu fermentation. It also is important to control the urea and EC contents in Hongqu Huangjiu production.

## Introduction

There are potential safety risks around ethyl carbamate (EC) in Hongqu Huangjiu production (Luo et al., 2017; Zhou et al., 2020). EC is produced in trace amounts during the fermentation or aging of many fermented foods and alcoholic beverages and is a likely carcinogen to humans (i.e., class 2A) (Xian et al., 2019). Indeed, 90% of EC in rice wine is produced by the reaction of the urea and the alcohol of *Saccharomyces cerevisiae* (Vázquez et al., 2017; Fang et al., 2018). Hongqu Huangjiu is a traditional rice wine in China (Huang et al., 2019); “Hong Qu” (a traditional Chinese fermentation starter) has a unique flavor and rich functional nutrients (Lv et al., 2015; Huang et al., 2018; Liang et al., 2020a). However, it also has a high EC safety risk. Our previous studies found that Hongqu Huangjiu contained rich amino acids such as arginine, which are precursors of urea production (Liang et al., 2020b). If the urea production level is not targeted repression, then there could be higher EC content in Hongqu Huangjiu production. This has harmed the development of the Hongqu Huangjiu industry.

There are currently several common methods to control urea content. (1) Urease can degrade the urea content. Urease is often added to hydrolyze the urea during the production of grape wine and Japanese sake (Yang et al., 2019). The urea content in rice wine could be reduced by more than 90% using urease (Liu et al., 2019). However, urease quickly loses activity, and its use can dramatically increase production costs. (2) Rice wine yeast can be screened for low urea production. For example, *Saccharomyces cerevisiae* mutant strains with low urea production levels (14.91 mg/L and 17.89 mg/L) were screened with He-Ne laser radiation and nitroso arc combined with mutagenesis, UV, and γ-ray combined mutagenesis (Kuribayashi et al., 2017). (3) Low-yield urea *Saccharomyces cerevisiae* can be engineered. A non-urea-producing *Saccharomyces cerevisiae* strain was obtained by knocking out the arginase gene *CAR1* in sake yeast (Yoshiuchi et al., 2000). *Saccharomyces cerevisiae* strain with low urea production was constructed by over-expressing the intracellular urea amidase genes *DUR1* and *DUR2* (Wu et al., 2016). However, most of these strains are mutagenesis and engineering strains with potential problems in food safety.

In our previous experiments, we screened and obtained a *S. cerevisiae* strain JH301 that had lower urea production. However, the key genes responsible for low urea production of strain JH301 have not yet been researched; the molecular mechanism of low urea production of strain JH301 remains unclear. Here, we assessed the whole genome of strain JH301 using a high-throughput sequencing technology in MiSeq from Illumina and long-read sequencing technology in a PacBio Sequel platform. We focused on the key genes in strain JH301 using comparative genomics with *Saccharomyces cerevisiae* model strain S288c. Finally, we used PCR and Quantitative real-time PCR (qRT-PCR) analyses to verify the key genes from DNA and RNA levels. The results could guide the screening of similar yeasts and provide a basis for molecular breeding of yeasts with low urea production. This is also useful for a scientific understanding of the mechanism of low urea production by *Saccharomyces cerevisiae* during Hongqu Huangjiu fermentation and it is important to control the urea and EC contents in Hongqu Huangjiu production.

## Materials and Methods

### Strain Material and Mediums

*Saccharomyces cerevisiae* strains JH301, JH303, JH401, JH405, and JH505 are usually used for Hongqu Huangjiu production; both were isolated from Wuyi Hongqu starters in the Fujian Province of China. *Saccharomyces cerevisiae* strain AQ was provided by a wine company (Fujian, China). *Saccharomyces cerevisiae* strain S288c was obtained from NITE Biological Resource Center.

### Activation of *Saccharomyces Cerevisiae* Strains

The *Saccharomyces cerevisiae* strains were precultured on YPD plates at 25^°^C for 24 h. A single colony was activated in 50 mL YPD medium (25^°^C, 200 rpm). The growth was monitored by determining the optical density at 600 nm (OD 600).

### Determination of the Content of Alcohol, Urea, and Ethyl Carbamate of *Saccharomyces cerevisiae* Strains

Here, 5% activation liquid of *Saccharomyces cerevisiae* strain was mixed with 200 mL rice liquid medium. The medium underwent fermentation at 25^°^C for 20 days. Solid-liquid separation was performed, and the fermented liquid was centrifuged to obtain the supernatant for future alcohol, urea, and EC content determination. Full evaporation headspace gas chromatography measurements were used to assess the content of alcohol as previously described (Liang et al., 2020c). The urea content was measured by high-performance liquid chromatography (Clark et al., 2007). The EC content in Hongqu Huangjiu was analyzed using solid-phase extraction and stable isotope dilution GC/MS (Lachenmeier et al., 2006). The urea and EC contents were determined in each sample after dilution to 15% alcohol. Each group of liquids was fermented using the same *Saccharomyces cerevisiae* strain in three independent experiments.

### Whole Genome Sequencing Analysis of *Saccharomyces cerevisiae* Strains

A total of four PacBio 20-kb libraries were generated with a SMRTbell Template Prep Kit (PacBio), and the libraries were sequenced on the PacBio Sequel platform. A total of 72 Gb (∼124×) of PacBio sequence data were generated. A total of ∼113 Gb (∼194 × fold coverage) of 10X Chromium library data were sequenced on an Illumina MiSeq system with paired-end 150-bp reads. The RNA library preparation, sequencing, genome assembly, genome annotation, and quality assessment were performed by Shanghai Personal Biotechnology Co., Ltd. (Shanghai, China). Function annotation was completed by BLAST search against KEGG (Kyoto Encyclopedia of Gene and Genomes) database (Moriya et al., 2007).

### Comparative Genome Analysis

Through collinearity analysis between the two genomes, we can observe the insertion and deletion of sequences between the target species genome and the reference genome. Such analysis confirms the local positional arrangement relationship and finds the regions of translocation, inversion, and translocation + inversion. The software Mummer (version 3.23) and Mauve (version 2.3.1) were used to conduct a rapid comparison between the two genomes, determine the relative direction of the sequence, and adjust the genome to align the starting point of the genome. Chainnet software was used to join the comparison results into longer comparisons. LastZ (Version 1.03.54) software was used to detect the indels (Shalhub et al., 2014) with a length of less than 50 bp. A database based on the data set was constructed and used as a query to perform all-VS-all BLASTP analysis. The result of sequence alignment was processed with OrthoMCL (version 2.0.8) software (Fischer et al., 2011); the length of sequence alignment was set to 70%, and MCL was used to cluster gene families. Finally, Perl script was used to sort and count the results of the clustering. Unique or missing genes were identified.

### DNA Extraction and PCR Conditions

*Saccharomyces cerevisiae* strain cells were pre-grown in YPD at 28^°^C for 48 h. The yeast DNA was extracted from the pure cultures using the method described by Duarte (Duarte et al., 2009). The 25-μL PCR mixture contained 12⋅5 μL de Mix GoTaq^®^ Green Master 2X (omega), 2 μL of DNA diluted to 10 ng/μL and 0.5 μmol/L of each primer. The sequences of the primers (De Melo Pereira et al., 2010) utilized in this study and the amplification conditions are shown in Table 1. Amplification products were separated by electrophoresis on a 0.8% (w/v) agarose gel and stained with SYBR Green (Invitrogen, United States). DNA fragments were visualized by UV transillumination, and images were captured using a Polaroid camera. A ladder marker was used as a size reference.

**TABLE 1:** List of PCR primer pairs designed and used for this study.

Primers	Sequence	Amplification conditions
*ARG80*	5’-CGAATAGCGACGGTTCAAGT-3’; 5’-CGTCAGGAGTGTCTGAAGCA-3’	35 cycles 94^°^C 30 s/61^°^C 30 s/72^°^C 2 min
*BIO3*	5’-CATATACACCAGATGTCGCG-3’; 5’-CCTTCGTCAATTCCTCTGTC-3’	35 cycles 94^°^C 30 s/61^°^C 30 s/72^°^C 2 min
*VTC4*	5’-GGTGAGCACTTGAGCAAGTC-3’; 5’-AAACACAGAAACCATGCCGG-3’	35 cycles 94^°^C 30 s/61^°^C 30 s/72^°^C 2 min

### Quantitative Real-Time PCR Analysis

#### RNA Extraction

The total RNA samples were isolated by TRIzol reagent (TIANGEN BIOTECH, Beijing). The RNA purity and concentration were then measured using a NanoPhotometer spectrophotometer (IMPLEN, CA, United States).

#### Quantitative Real-Time PCR Analysis

cDNA was synthesized using 2 μg RNA using a PrimeScript™ RT reagent Kit with gDNA Eraser (TaKaRa). Gene-specific primers for quantitative real-time PCR (qRT-PCR) analysis were designed using Primer 5.0 by Allwegene Technology (Allwegene Technology Co., Ltd., Beijing, China). The QACT gene was used as a house-keeping gene. qRT-PCR reaction was performed using SYBR^®^ Premix Ex Taq™ II (Tli RNaseH Plus) on an ABI 7500 real-time Detection System (Thermo Fisher Scientific, United States). The PCR reaction was conducted with the following reaction conditions: 95^°^C for 30 s, followed by 45 cycles of 95^°^C for 5 s, and 60^°^C for 40 s. The sequences of the primers utilized in this study are shown in Table 2. Samples for qRT-PCR were run in three biological replicates, with three technical replicates, and the data were represented as the mean ± *SD* (*n* = 3) for Student’s *t*-test analysis. The relative gene expression was calculated using the 2^–△△^^CT^ algorithm (Pfaffl, 2001).

**TABLE 2:** List of QPCR primer pairs designed and used for this study.

Primers	Sequence	Product size (bp)
*ARG80*	5’-CAACTCCCACCCCAACCATA-3’; 5’-GGCGTTGTGAAAGTGTAGACCAG-3’	232
*BIO3*	5’-AGGATTTGGTAGAACAGGTG-3’; 5’-AGTAGGGCTATTTGGAGAAG-3’	208
*VTC4*	5’-AGTTTGGTGAGCACTTGAGC-3’; 5’-CGTCCACTGACCGTTATTCT-3’	119
QACT	5’-TATGGAAAAGATCTGGCATCA-3’; 5’-CGGTTTGCATTTCTTGTTCG-3’	205

*Primer QACT is the primer of house-keeping gene.*

### Statistical Analysis

All experiments were performed in triplicate. Data are represented as means ± standard deviation (± s). Statistical analyses were performed using SPSS 15.0 software using one-way analysis of variance, and *P* < 0.05 indicates statistical significance.

## Results

### Urea and Ethyl Carbamate Production Levers of Different *Saccharomyces cerevisiae* Strains

The urea and EC production levers of different *Saccharomyces cerevisiae* strains were measured by HPLC and SPE-GC/MS analysis, respectively, as shown in Table 3. There were significant differences in the levels of urea and EC production among strains. The urea and EC production levers of *S. cerevisiae* strain JH301 was the lowest. Compaired with strain S288c, the urea content of JH301 was decreased by 69.32%, the EC content at baseline was decreased by 51.14%, and the EC content after 6 months decreased by 46.82%, respectively. The results showed that strain JH301 had a lower urea and EC production levers than other strains in this study.

**TABLE 3:** Comparison of fermentation quality indexes of different *Saccharomyces cerevisiae* strains.

*Saccharomyces cerevisiae* strains	JH301	JH303	JH401	JH405	JH505	S288c
Alcohol (%)	16.67 ± 0.47^a^	14.77 ± 0.38^c^	14.46 ± 0.15^c^	16.44 ± 0.23^a^	15.25 ± 0.18^b^	15.53 ± 0.68^b^
Urea content (mg/L)	16.33 ± 1.15^c^	55.22 ± 3.19^a^	33.54 ± 1.64^b^	36.22 ± 2.82^b^	35.47 ± 1.42^b^	53.23 ± 3.19^a^
EC content at baseline (μg/L)	53.25 ± 2.80^c^	113.87 ± 7.23^a^	89.49 ± 4.28^b^	85.82 ± 3.47^b^	85.82 ± 6.93^b^	108.99 ± 10.42^a^
EC content after 6 months (μg/L)	70.66 ± 3.69^c^	135.38 ± 4.28^a^	101.84 ± 5.31^b^	104.48 ± 7.14^b^	105.19 ± 6.22^b^	132.88 ± 8.46^a^

*JH301 fermented using Saccharomyces cerevisiae strain JH301; S288c fermented using Saccharomyces cerevisiae strain S288c; JH303, JH401, JH405, and JH505 fermented using Saccharomyces cerevisiae strain JH303, JH401, JH405, and JH505, respectively. The urea and EC content were determined in each sample. Data points represent the means of three biological repeats, and error bars represent standard deviations. Significant difference shown as lowercase letters means the significance level was lower than 0.05 (P < 0.05).*

### Whole Genome Sequencing Analysis of *Saccharomyces cerevisiae* Strain JH301

The whole genome sequencing of *S. cerevisiae* strain JH301 was accomplished using the PacBio Sequel and Illumina MiSeq platforms. The sequencing quality data included sequence base quality by next-generation high-throughput sequencing technology (Supplementary Figure 1), length of reads by the long-read sequencing technology (Supplementary Figure 2 and Supplementary Table 1), and assembly integrity of the whole genome of strain JH301 (Supplementary Table 2). The results of *de novo* assembly showed that the total sequence length of 11.97 Mb was predicted to contain 5,228 predicted genes in *S. cerevisiae* strain JH301. The genome was predicted to comprise 16 chromosomes by pairwise comparison against the chromosomes of the public reference strain S288c from the National Center for Biotechnology Information (NCBI). The genome characteristics of strain JH301 had considerable differences with S288c for genome parameters that include total sequence length. In addition, the number of rRNA in strain JH301 was 6.6-fold higher than strain S288c. Table 4 summarizes the whole genome sequencing and annotation results. This whole genome shotgun project has been deposited at GenBank under the accession JALDNA000000000.

**TABLE 4:** Whole genome sequencing and annotation results.

Property	JH301	S288C
Total sequence length (Mb)	11.97	12.16
GC content (%)	38.02	38.15
Genes percentage of genome (%)	67.07	75.37
Predicted genes number	5,228	5,906
Average gene length (bp)	1536.5	1,369
The number of tRNA	297	299
The number of rRNA	66	10

*JH301 fermented using Saccharomyces cerevisiae strain JH301; S288c fermented using Saccharomyces cerevisiae strain S288c.*

**FIGURE 1:**
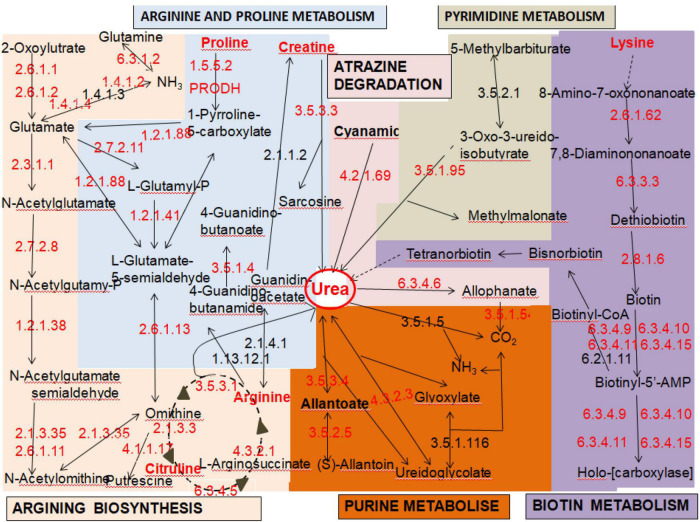
Pathways related to the urea metabolism of *S. cerevisiae* strain JH301.

Among the 5,228 predicted genes in the entire strain genome, 3,155 (63.85%) genes could be annotated according to the KEGG database. These genes could be classified into five gene functional annotations: genetic information processing (2,237 genes), metabolism (1,421 genes), cellular processes (710 genes), environmental information processing (521 genes), and organismal systems (495 genes). Based on KEGG pathway mapping, we annotated 346 pathways in the JH301 genome. Of these, there are six main pathways related to urea metabolism. The overall response pathways are shown in Figure 1. (1) map00220: arginine biosynthesis (most common pathway of urea metabolism), (2) map00230: purine metabolism, (3) map00240: pyrimidine metabolism, (4) map00330: arginine and proline metabolism, (5) map00780: biotin metabolism, and (6) map00791: atrazine degradation. The results showed that strain JH301 could convert amino acids such as arginine, citrulline, lysine, proline, and ornithine to produce urea. It also had urea carboxylase (EC6.3.4.6), urease (EC3.5.3.4), and ureidoglycolate lyase (EC4.3.2.3) to decompose urea.

### Analysis of Key Genes Responsible for Low Urea Production of Strain JH301

**FIGURE 2:**
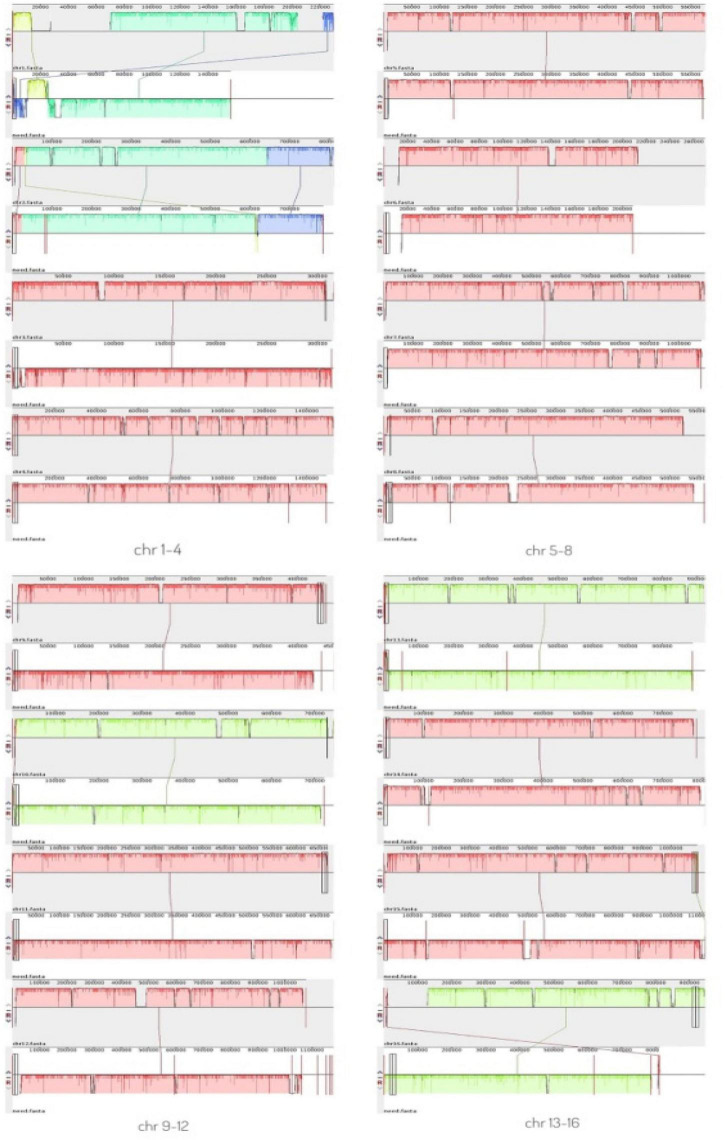
Mauve analysis on 16 chromosomes between the genome of strains JH301 and S288c. The top bar shows the chromosome of strain JH301; the bottom bar shows the chromosome of strain S288c.

**FIGURE 3:**
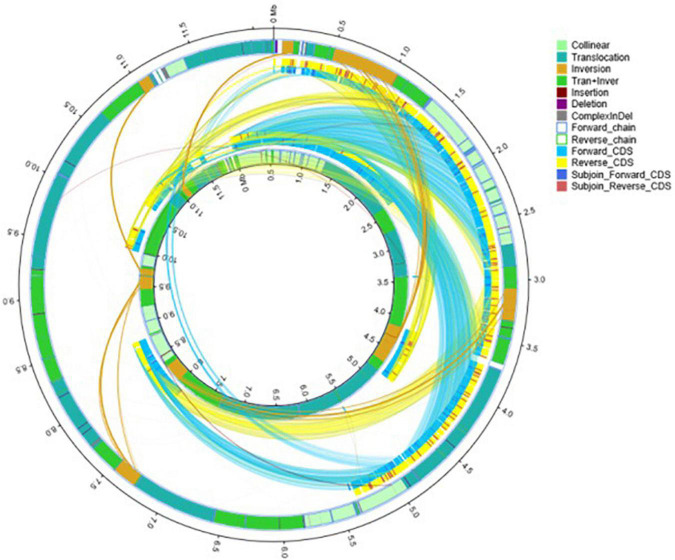
Detailed circular genome diagram between the genomes of strains JH301 and S288c. The outer circle denotes the characteristics of the entire genome of strain JH301, and the inner circle denotes strain S288c.

**FIGURE 4:**
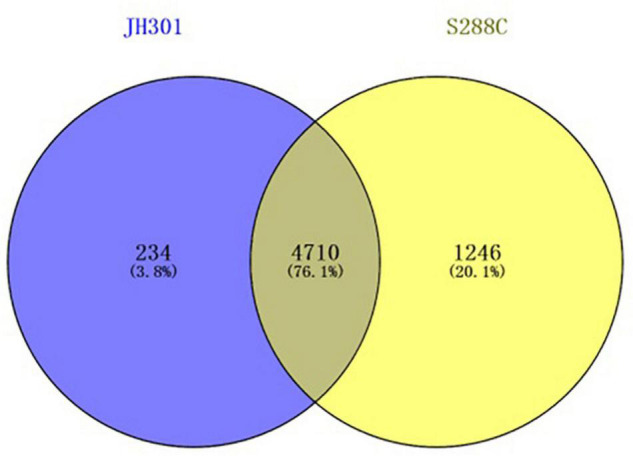
Venn analysis between the genomes of strains JH301 and S288c.

A comparative genomic analysis between *S. cerevisiae* strain JH301 and the model *S. cerevisiae* strain S288c was performed using MUMmer, Mauve, and OrthoMCL software. Figure 2 compares the differential genes on 16 chromosomes between the genome of JH301 and S288c using the Mauve analysis. A detailed circular genome diagram between the genome of JH301 and S288c is shown in Figure 3. A total of 60,885 SNPs (53,933 homozygous SNPs, 6,952 heterozygous SNPs) and 5,412 InDels (<50 bp, 4,391 homozygous InDels, 1,021 heterozygous InDels) were detected. By annotating SNPs and InDels, we found 12,518 non-synonymous mutations and 25,551 synonym mutations in SNPs. There were 95 mutations leading to frameshift insertion (product extension) and 98 mutations leading to frameshift deletion (product shortening) in InDels. These results indicated that there were two types of chromosomal structural variations between the genome of JH301 and S288c. One type is a lack of fragments. The other is an increase in fragments. To identify species-specific genes, we performed pairwise comparisons using a series of All-VS-All BLASTP searches between the two strains in KEGG database and ‘‘PANTHER’’ Classification System.^1^ Figure 4 shows 4,770 genes identified based on sequence similarities: 234 genes and 1,246 genes were species-specific in *S. cerevisiae* strains JH301 and S288c, respectively.

We next performed KEGG pathway mapping of the species-specific genes. There were three species-specific genes referred to the urea production pathway that missed in strain JH301 but involved in S288c via comparative genomic analysis of the lack and increase of fragments. Three species-specific genes included the following: (1) The *ARG80* gene (NC_001145.3; in Chromosome: XIII) is for arginine metabolism regulation protein I (K19808). It is a key protein for pathway of arginine biosynthesis (map00220). (2) The *BIO3* gene (NC_001146.8; in Chromosome: XIV) is for adenosylmethionine-8-amino-7-oxononanoate aminotransferase protein (K00833), which is a key protein in the pathway of biotin metabolism (map00780). (3) The *VTC4* gene (NC_001142.9; in Chromosome: X) is for proline dehydrogenase protein (K00318) which is a key protein in the pathway of arginine and proline metabolism (map00330).

### Verification of Key Genes Responsible for Low Urea Production of Strain JH301

**FIGURE 5:**
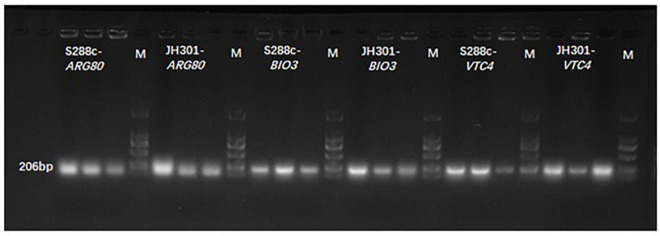
Banding patterns of the PCR products. M means marker; S288c-*ARG80* means the PCR bands of *ARG80* gene of strain S288c; JH301-*ARG80* means the PCR bands of *ARG80* gene of strain JH301; S288c-*BIO3* means the PCR bands of *BIO3* gene of strain S288c; JH301-*BIO3* means the PCR bands of *BIO3* gene of strain JH301; S288c-*VTC4* means the PCR bands of *VTC4* gene of strain S288c; JH301-*VTC4* means the PCR bands of *VTC4* gene of strain JH301.

We further conducted PCR analysis of key genes to verify them at the DNA level. Figure 5 shows that strain JH301 is not completely missing from these three genes. This is probably because these three genes were also identified at other chromosomes along the whole genome of strain JH301. Therefore, the total expression levels of these three genes were further investigated by qRT-PCR to verify the key genes from the RNA level (Capece et al., 2016). Table 5 show quantitative expression levels of these three genes investigated by quantitative real-time PCR vs. strain S288c, the expression levels of the *ARG80* gene in strain JH301 were significantly reduced 40.09 times (*P* < 0.05); and the expression levels of the other two genes were not significantly different (*P* > 0.05). The results showed that *ARG80* gene is likely to be the key gene responsible for low urea production. This gene had low expression and could restrict the expression of arginine metabolism regulation protein. Therefore, it should likely limit the pathways whereby arginine is converted to the urea in strain JH301.

**TABLE 5:** Quantitative real-time PCR analysis results.

Gene name	Gene description	Sequence	Strain JH301	Strain S288c
*ARG80*	K19808	Chromosome: XIII; NC_001145.3	1.00 ± 0.05b	40.09 ± 0.32a
*BIO3*	K00833	Chromosome: XIV; NC_001146.8	1.06 ± 0.09a	1.11 ± 0.19a
*VTC4*	K00318	Chromosome: X; NC_001142.9	1.06 ± 0.32a	0.99 ± 0.39a

*JH301 fermented using Saccharomyces cerevisiae strain JH301; S288c fermented using Saccharomyces cerevisiae strain S288c. Data represent the means of three biological replicates. Significant difference shown as lowercase letters means the significance level was lower than 0.05 (P < 0.05).*

## Discussion

Next-generation high-throughput sequencing technology combined with long-read sequencing technology such as Pacific Biosciences (PacBio) can complete whole genome analysis of yeast (Goodwin et al., 2016). These data can mine key functional genes of *S. cerevisiae* vs. *S. cerevisiae* species model strain S288c (Baert et al., 2021). For example, some researchers have used this technique to study the whole genome of yeast Cen.PK113-7D and *Saccharomyces cerevisiae* S288c was used as the control to investigate functional genes related to the fermentation process in the strain Cen.PK113-7D (Jenjaroenpun et al., 2018). The key genes of low acetaldehyde production in brewer’s yeast M14 were studied and compared to the S288c genome, and the molecular mechanism of low acetaldehyde production in this strain was described (Liu et al., 2018). The key genes of high ethanol tolerance in *Saccharomyces cerevisiae* YF17 were analyzed using *Saccharomyces cerevisiae* S288c and Japanese sake strain K7 as a control (Li et al., 2014). The differences between *Saccharomyces cerevisiae* N85 and sake yeast K7 in rice wine were also analyzed (Zhang et al., 2018). Therefore, we used the whole genome sequencing and comparative genomic analysis in this study and found that *ARG80* gene was the key gene responsible for low urea production of *S. cerevisiae* JH301 for the first time.

**FIGURE 6:**
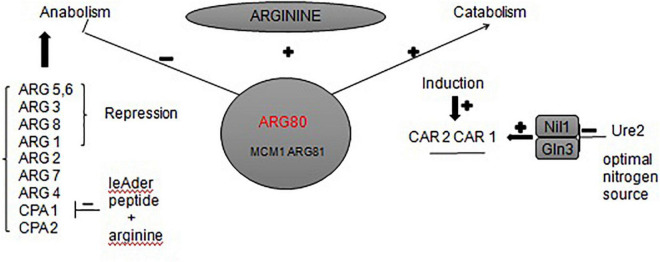
Network of regulatory circuits controlling the arginine anabolic and catabolic genes in response to nitrogen signals.

To the best of our knowledge, the urea metabolic pathway of *Saccharomyces cerevisiae* in rice wine mainly focuses on the arginine metabolic pathway (map00220 Arginine biosynthesis). Arginine is a conventional nitrogen source used by *Saccharomyces cerevisiae*. During the growth and reproduction of *Saccharomyces cerevisiae*, a large amount of urea is synthesized through argininase *car1* (Wu et al., 2014). It satisfies its own needs for growth, but the excess urea is secreted into the fermentation broth (Lee et al., 2017). When the utilization of ammonia is complete, *Saccharomyces cerevisiae* will start to use urea as a nitrogen source; urea is then decomposed into ammonia and carbon dioxide by the urea-amidase *DUR1* and *DUR2* (Gowd et al., 2018). Other studies found alternative key genes in the arginine biosynthesis pathway. For example, the expression of arginine transporter gene *VBA2* could be significantly up-regulated by adding bamboo leaf extract during fermentation (Zhou et al., 2020). The expression of arginine deiminase gene *adi* could be inhibited by added gallic acid and protocatechuic acid (Zhou et al., 2017). These both inhibited the transformation of arginine to produce urea. In this study, we found that the *ARG80* gene is also the key gene responsible for urea production of *S. cerevisiae* JH301. The *ARG80* gene is an important gene for the transport of arginine to yeast cells and is one of the four regulatory genes in the regulation of arginine metabolism (Kloosterman and Kuipers, 2013). Figure 6 summarizes different regulations involved in the control of the expression of arginine anabolic and catabolic genes. *ARG80*, *ARG81*, and *MCM1* could form a complex interacting with DNA sequences called »arginine boxes« present in the promoters of arginine co-regulated genes (Messenguy and Dubois, 1999). In the presence of arginine, they are required to repress the synthesis of five anabolic enzymes and to induce the synthesis of two catabolic enzymes (Jamai et al., 2002). If *ARG80* gene had low expression, it could restrict the activity of arginine metabolism regulation protein. Therefore, this gene is likely one of the main reasons for the low urea production of strain JH301 during Hongqu Huangjiu fermentation. Further studies can verify the function of this gene through construction of new engineering strains such as *ARG80* gene overexpression in strain JH301 and *ARG80* gene knockout in strain S288c.

Ethanol reacts with the urea produced during metabolism of arginine to give EC. Previous studies have reported that the formation of EC in wine was correlated to the concentration of urea, and the concentration increased substantially with increasing temperature (Weber and Sharypov, 2009). Increased EC content during fermentation increases the risk of cancer through consumption of alcoholic beverages (Jiao et al., 2014). Therefore, it is imperative to control urea content to control EC content. Further research will focus on regulating the expression of the key gene *ARG80* to decrease the urea content during Hongqu Huangjiu fermentation. This step is useful to control the quality and safety of Hongqu Huangjiu.

## Conclusion

In conclusion, we assessed the whole genome of *S. cerevisiae* strain JH301 using next-generation high-throughput sequencing technology in MiSeq from Illumina and the long-read sequencing technology in PacBio Sequel platform. We also found that gene *ARG80* is likely to be responsible for the low urea production of strain JH301 for the first time using comparative genomics analysis vs. *Saccharomyces cerevisiae* model strain S288c. Finally, we used PCR and qRT-PCR analyses to verify the key genes from DNA and RNA levels. Further studies can verify the functions of this gene via gene overexpression and gene knockout techniques. The results are important to control the urea and EC contents in Hongqu Huangjiu production.

## Data Availability Statement

The datasets presented in this study can be found in online repositories. This whole genome shotgun project has been deposited at GenBank under the accession JALDNA000000000.

## Author Contributions

ZL and ZH: conceptualization. ZL, HS, XR, XZL, and XYL: methodology. ZL and HS: software. ZL, XZL, and ZH: validation. ZH: formal analysis, writing—review, and editing. XZL: investigation. ZL and YZ: data curation. ZL: writing—original draft preparation and visualization. All authors have read and agreed to the published version of the manuscript.

## Conflict of Interest

XYL was employed by Fujian Pinghuhong Biological Technology Co., Ltd. The remaining authors declare that the research was conducted in the absence of any commercial or financial relationships that could be construed as a potential conflict of interest.

## Publisher’s Note

All claims expressed in this article are solely those of the authors and do not necessarily represent those of their affiliated organizations, or those of the publisher, the editors and the reviewers. Any product that may be evaluated in this article, or claim that may be made by its manufacturer, is not guaranteed or endorsed by the publisher.

## Footnotes

1
http://www.pantherdb.org/
